# Te Nanoneedles Induced Entanglement and Thermoelectric Improvement of SnSe

**DOI:** 10.3390/ma13112523

**Published:** 2020-06-01

**Authors:** Hyun Ju, Myeongjin Kim, Jinglei Yang, Jooheon Kim

**Affiliations:** 1 School of Chemical Engineering & Materials Science, Chung-Ang University, Seoul 06974, Korea; mohani@cau.ac.kr; 2 Department of Hydrogen & Renewable Energy, Kyungpook National University, 80 Daehakro, Bukgu, Daegu 41566, Korea; 3 Department of Mechanical and Aerospace Engineering, The Hong Kong University of Science and Technology, Hong Kong SAR, China

**Keywords:** thermoelectric, tellurium, nanoneedles, inner-site crystallization, tin selenide

## Abstract

Chalcogenide-based materials have attracted widespread interest in high-performance thermoelectric research fields. A strategy for the application of two types of chalcogenide for improved thermoelectric performance is described herein. Tin selenide (SnSe) is used as a base material, and Te nanoneedles are crystallized in the SnSe, resulting in the generation of a composite structure of SnSe with Te nanoneedles. The thermoelectric properties with various reaction times are investigated to reveal the optimum conditions for enhanced thermoelectric performance. A reaction time of 4 h at 450 K generated a composite Te nanoneedles/SnSe sample with the maximum *ZT* value, 3.2 times larger than that of the pristine SnSe. This result is attributed to both the reduced thermal conductivity from the effective phonon scattering of heterointerfaces and the improved electrical conductivity value due to the introduction of Te nanoparticles. This strategy suggests an approach to generating high-performance practical thermoelectric materials.

## 1. Introduction

Tin selenide (SnSe) is an interesting example of chalcogenide-based materials and has been widely studied in a range of research areas including chemistry, electronics, and metallurgy. It has also potential as a high-performance thermoelectric material because of its large Seebeck coefficient (*S*) value [1] (higher than previously reported values for commonly applied thermoelectric tellurides [2,3,4,5] and low thermal conductivity (*κ*) value, which leads to the excellent thermoelectric performance [1]. The thermoelectric performance is indicated by the dimensionless figure of merit (*ZT*) given by (*σ*·*S*^2^·*T*)/*κ*. The parameters, *σ*, *T*, and *κ* indicate the electrical conductivity, absolute temperature, and thermal conductivity, respectively. Several examples relating to the significant enhancement of *ZT* in SnSe have been reported previously [6,7,8,9,10]. However, the further enhancement of *ZT* for SnSe remains important in practical thermoelectric applications.

Te is another elemental material in the chalcogenide group that is also widely studied. Recent advances in nanotechnology have provided a range of techniques for the synthesis of Te in the form of low-dimensional nanostructures with the potential to replace bulk materials due to their outstanding material properties [11,12,13,14,15]. For example, the bacterial synthesis of tellurium nanostructures has been reported by Wang et al. [16], while Amani et al. described the fabrication of air-stable Te nanoflakes for photodetectors via solution synthesis [17]. Nanostructured Te has been also studied for application in high-performance thermoelectric materials with high *σ* and *S*. For example, Qiu et al. reported the preparation and excellent thermoelectric performance of two-dimensional nanostructured Te [18].

In the present paper, a promising approach to enhancing the thermoelectric performance of SnSe is described. One-dimensional (1D) needle-like Te nanostructures were fabricated by a solution-based synthetic method. Te^2–^ anions were located near the surface of SnSe, and 1D growth of Te was observed after nucleation of the ions due to the intrinsic anisotropy of Te. Interestingly, Te nanoneedles were formed on the surface of SnSe, resulting in the fabrication of a Te nanoneedles/SnSe composite structure. The thermoelectric properties of the resulting product were examined to identify potential thermoelectric applications. It is anticipated that the combination of Te nanoneedles and SnSe can provide an efficient thermoelectric composite material.

## 2. Results and Discussion

**Figure 1:**
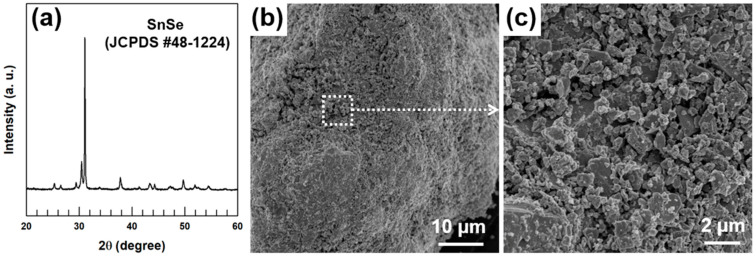
Results of (**a**) XRD analysis, (**b**) low-, and (**c**) high-magnification FE-SEM imaging of the SnSe powder.

The SnSe bulk material was pulverized to a fine powder and the product was investigated by a X-ray diffraction (XRD, New D8-Advance, Bruker-AXS, Pangyo, Korea). Figure 1a presents the XRD pattern of the as-milled SnSe powder, revealing the diffraction peaks typically ascribed to the orthorhombic structure of the Pnma crystal system (JCPDS # 48-1224) [19,20,21]. The pure crystalline phase of SnSe was observed with no impurity peaks. The powder product was further characterized by field-emission scanning electron microscopy (FE-SEM, SIGMA, Oberkochen, German) imaging (Figure 1b,c). Numerous micrometer-sized particles were observed in the low- and high-magnification FE-SEM images.

**Figure 2:**
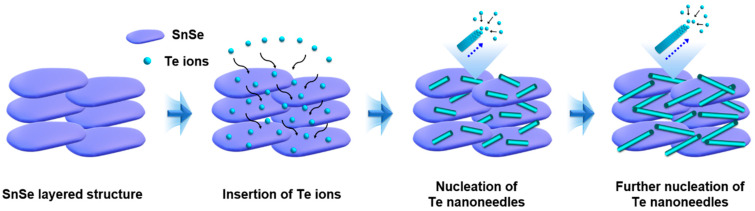
Schematic mechanism for the fabrication of Te nanoneedles/SnSe composites.

The mechanism for the fabrication of the Te nanoneedles/SnSe composites is shown schematically in Figure 2. The SnSe particles are randomly distributed in the ethylene glycol solution. The TeO_2_ is then dissolved in the solution to provide numerous Te nuclei. The growth of Te nanoneedles from the Te^2–^ proceeds via nucleation and subsequent anisotropic 1D growth, according to their intrinsic growth tendency [22]. The majority of Te nanoneedles are formed near the SnSe because the crystallization of Te^0^ takes place preferentially on the substrate (SnSe) rather than in the solution phase [23]. While some of the ions are positioned on the SnSe surface, a great many ions are introduced between the SnSe under the high temperature condition (433 K) in solution phase, resulting in the crystallization of Te nanoneedles.

**Figure 3:**
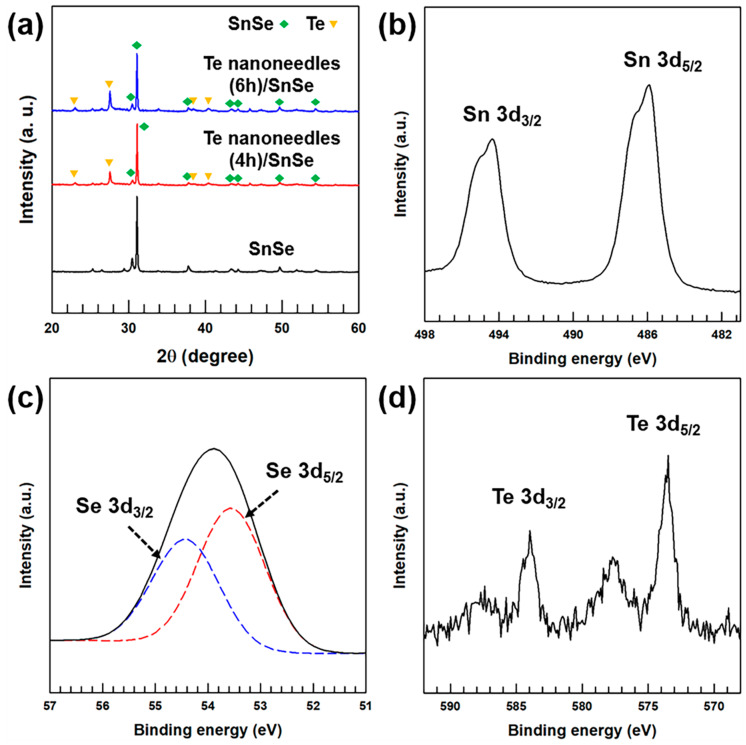
(**a**) XRD patterns of Te nanoneedles/SnSe composites with various reaction times; (**b**–**d**) XPS core spectra of (**b**) Sn, (**c**) Se, and (**d**) Te regions for the Te nanoneedles(4 h)/SnSe composite.

The crystalline phases of the Te nanoneedles(x)/SnSe composites were examined by XRD. The XRD patterns of the materials produced at various reaction times (Figure 3a) indicate that all the samples display the same SnSe diffraction peaks, while the intensity of Te peaks (Figure S1) enhances with increasing the reaction time. This suggests the presence of different contents of Te crystals in the SnSe-based samples. The composite samples were further examined by X-ray photoelectron spectroscopy (XPS) in order to identify the binding energies. The survey scan XPS spectrum of the Te nanoneedles(4 h)/SnSe composite is shown in Figure S2, revealing various peaks including Sn, Se, and Te. The high-magnification XPS core-level spectrum of the Sn 3d region (Figure 3b) displays peaks at ~485.8 and 494.3 eV, corresponding to the Sn 3d_5/2_ and Sn 3d_3/2_ binding energies, while the Se 3d spectrum (Figure 3c) displays peaks at ~53.5 and ~54.4 eV for Se 3d_5/2_ and Se 3d_3/2_ binding energies. For the Te 3d region, the binding energies at ~584.0 (Te 3d_3/2_) and 573.5 eV (Te 3d_5/2_) are observed. The binding energy values of the Sn, Se, and Te agree well with the previously reported values [21,24,25], further demonstrating the successful fabrication of the Te nanoneedles/SnSe composites.

**Figure 4:**
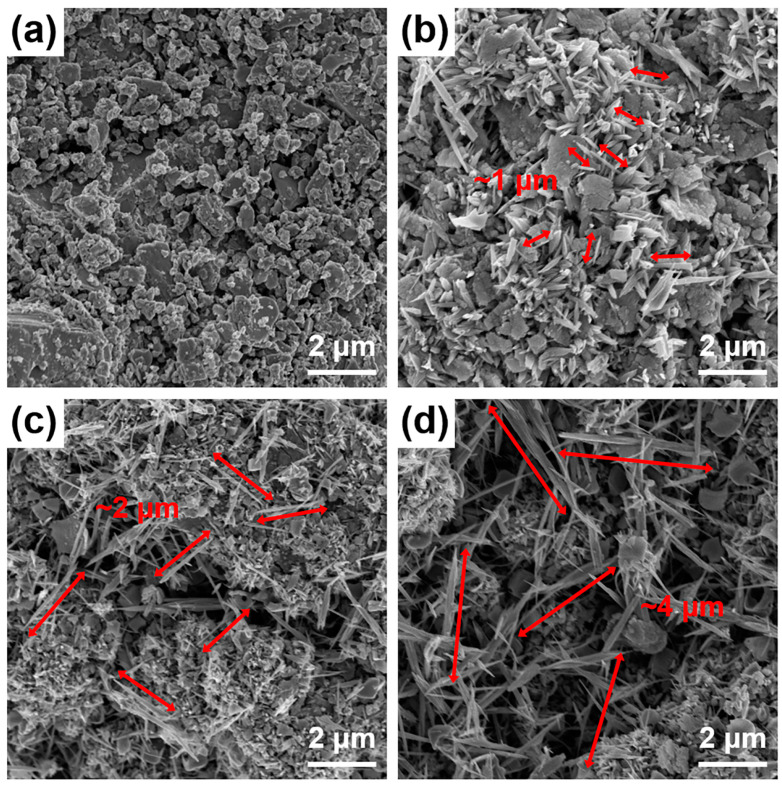
FE-SEM images of Te nanoneedles/SnSe composites with the reaction times of (**a**) 0 h, (**b**) 2 h, (**c**) 4 h, and (**d**) 6 h.

The above conclusions are supported by the FE-SEM images of the Te nanoneedles(x)/SnSe composite samples with various reaction times presented in Figure 4. The Te nanoneedles(2 h)/SnSe composite sample (Figure 4b) exhibits numerous nanostructured Te needles, which clearly differs from the pure SnSe sample (Te nanoneedles(0 h)/SnSe) in Figure 4a. The needle-like nanoparticles in the composite samples become longer as the reaction time is increased to 4 h (Figure 4c) and 6 h (Figure 4d). This result suggests that the prolonged reaction time leads to the crystallization of longer Te nanoneedles. Energy-dispersive X-ray spectroscopy (EDS) analysis of the Te nanoneedles(4 h)/SnSe composite was performed to further identify the existence and distribution of Te nanoneedles in the composites. Figure S3 shows the FE-SEM image of the Te nanoneedles(4 h)/SnSe composite and the resulting EDS mappings of Sn, Se, and Te atoms. The as-prepared Te nanoneedles showed strong intensities in the Te mapping and good distribution in the composite structure.

**Figure 5:**
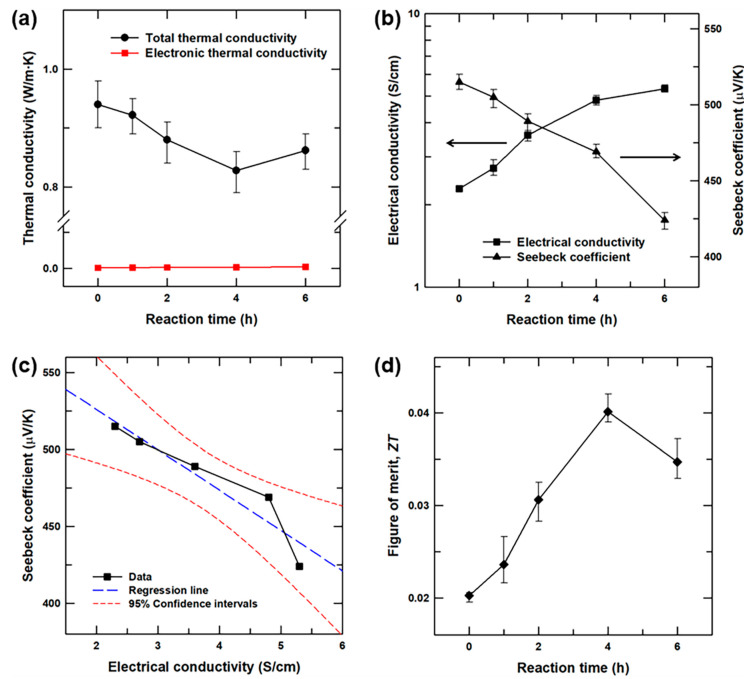
(**a**) Thermal conductivity (*κ*), (**b**) electrical conductivity (*σ*) and Seebeck coefficient (*S*), and (**d**) *ZT* values of the Te nanoneedles(x)/SnSe composites as a function of reaction time. (**c**) Seebeck coefficient values of the Te nanoneedles(x)/SnSe composites as a function of electrical conductivity.

The fabricated Te nanoneedles(x)/SnSe composites with various reaction times were pelletized to demonstrate the thermoelectric improvement under optimum reaction conditions. Figure 5a shows the *κ* and electronic contribution of thermal conductivity (*κ_e_*) values of the Te nanoneedles(x)/SnSe composite samples. The *κ* values of the samples were calculated, and the *κ_e_* values were estimated from the Wiedemann-Franz law, *κ_e_* = *L·T·σ*, where *L* indicates the Lorentz number (*L* = 2.45 × 10^−8^ W·Ω/K^2^) [26,27,28,29]. In Figure 5a, the dominant factor determining the *κ* values of the Te nanoneedles(x)/SnSe composites was the lattice scattering of phonons, because the *κ* was seen to be mainly independent of the *κ_e_* due to the relatively small contribution of the *κ_e_*. The introduction of Te nanoneedles into the SnSe resulted in the formation of heterointerfaces between the Te and SnSe, which acted as effective phonon scattering centers. Thus, the *κ* values of the Te nanoneedles(x)/SnSe composites were lower than that of pristine SnSe, in accordance with the previous reports on the phonon scattering in composite materials [30,31,32]. The *κ* value of the pristine SnSe was restricted efficiently by the scattering of phonons at the heterointerfaces for reaction times of up to 4 h. With reaction times greater than 4 h, the *κ* value subsequently increased as the higher volumetric fraction of Te nanoneedles in the composite (Table S1) enhanced the *κ* value due to the intrinsically higher *κ* value of Te relative to SnSe. The measured *σ* (left side) and *S* (right side) values of the Te nanoneedles(x)/SnSe composites with various reaction times are presented in Figure 5b. The *σ* value of the composites increases gradually with increasing reaction time, while the *S* value decreases in a trade-off relation (Figure 5c). This is because the Te nanoneedles create an electrically-conductive network but their *S* value is less than that of SnSe, so the Te nanoneedles interrupt the thermoelectrical transport in SnSe. The significantly enhanced power factor (*σ*·*S*^2^, Figure S4) and the reduced *κ* value of the Te nanoneedles(x)/SnSe composites led to enhanced *ZT* values, as indicated in Figure 5d. The maximum *ZT* value was achieved for the Te nanoneedles(4 h)/SnSe composite and was significantly larger than that of the pristine SnSe. To demonstrate the effect of material density on thermoelectric performance, the density (*ρ_d_*) values of the composites were investigated and the results were listed in Table S1. The *ρ_d_* decreases with increasing the reaction time, which is the proportional to the trend of the *S* and *κ* while it reveals inverse proportional trend to the *σ*. This result implies that the numerous formed heterointerfaces also effectively reduce the *ρ_d_* of composites, resulting in favorable effect of the reduction of *κ* and improved thermoelectric performance in the composite.

**Figure 6:**
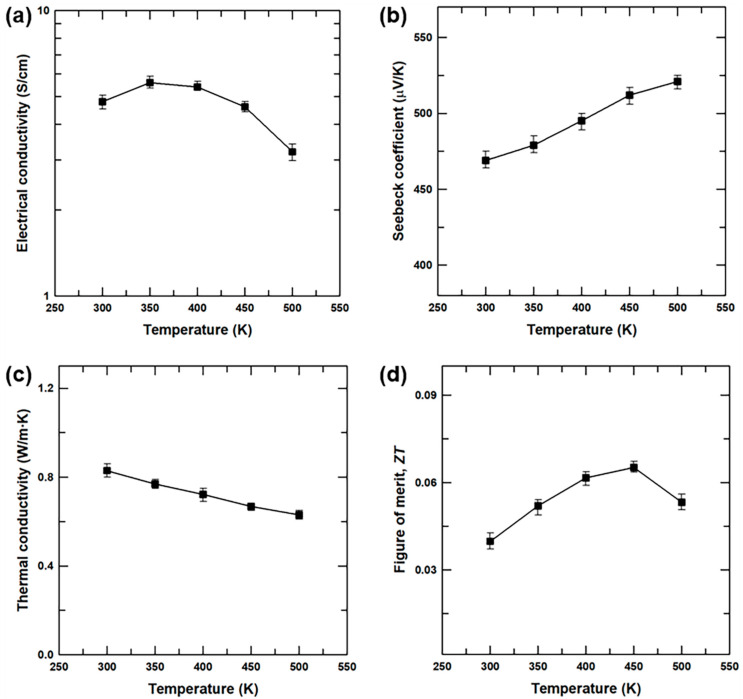
(**a**) Electrical conductivity (*σ)*, (**b**) Seebeck coefficient (*S*), (**c**) thermal conductivity (*κ*), and (**d**) *ZT* values of the Te nanoneedles(4 h)/SnSe composite sample as a function of a temperature.

The temperature dependence of the thermoelectric properties of the Te nanoneedles(4 h)/SnSe composite sample were also investigated. The temperature-dependent *σ* and *S* values presented in Figure 6a indicate metallic transport behavior in the measured temperature region that is in good agreement with the previous results for SnSe [7,33,34]. An initial decrease in the *σ* value with increasing temperature is again observed, and the *σ* value then increases at temperatures above ~400 K. In addition, the *S* value of the composite increases gradually with increasing the temperature (Figure 6b) while the *κ* value decreases with increasing temperature (Figure 6c). The calculated *ZT* value of the Te nanoneedles(4 h)/SnSe composite samples (Figure 6d) increases with increasing temperature to a maximum of 6.5 × 10^−2^ at 450 K, which is 3.2 times greater than that of the pristine SnSe. Hence, the hetero-structured product containing the Te nanoneedles in SnSe synergistically enhances the thermoelectric performance by increasing the *S* value and reducing the *κ* value.

## 3. Conclusions

In summary, the SnSe particles were pulverized by ball milling and distributed in the solution phase. A large quantity of Te nuclei were introduced into the SnSe surface under the high temperature condition (433 K) in solution, leading to the crystallization of 1D Te nanoneedles in SnSe. The reaction time was a critical factor in manipulating the morphologies of the 1D Te nanoneedles. In the visual images of the product, larger Te nanoparticles were observed in the Te nanoneedles/SnSe composites as the reaction time was increased. The fabricated Te nanoneedles(x)/SnSe composites with various reaction times were pelletized to demonstrate the thermoelectric improvement with optimization of the reaction conditions. The *κ* values of the Te nanoneedles(x)/SnSe composites were reduced because the introduction of Te nanoneedles into the SnSe resulted in the formation of heterointerfaces between the Te and SnSe, acting as effective phonon scattering centers. The *σ* values of the composites increased gradually with increasing reaction time, while the *S* value decreased. An enhanced thermoelectric *ZT* was achieved at the temperature of 450 K for the Te nanoneedles(4 h)/SnSe composite, which is 3.2 times greater than that of the pristine SnSe. The results of the present study demonstrated that the hetero-structured product of Te nanoneedles and SnSe could be widely used in a range of applications including high performance thermoelectric materials.

## Supplementary Materials

The following are available online at https://www.mdpi.com/1996-1944/13/11/2523/s1, Supporting Information Contents: 1. Experimental methods, 2. Figures, Figure S1: XRD patterns of the Te nanoneedles, Figure S2: Survey scan XPS spectrum of the Te nanoneedles(4 h)/SnSe composite, Figure S3: FE-SEM images of the Te nanoneedles(4 h)/SnSe composite and the resulting EDS mapping of Sn, Se, and Te atoms, Figure S4: Power factor (*σ·S*^2^) values of the Te nanoneedles(x)/SnSe composites as a function of reaction time.
