# Supplementary material for: Te Nanoneedles Induced Entanglement and Thermoelectric Improvement of SnSe
Source: Materials (Basel). 2020 Jun 1;13(11):2523. doi: 10.3390/ma13112523 (PMC7321485; doi:10.3390/ma13112523)
Supplement: Supplementary file 1 [file materials-13-02523-s001.pdf]

materials

Article

Te nanoneedles Induced Entanglement and Thermoelectric Improvement of SnSe

Hyun Ju [1], Myeongjin Kim [2,*], Jinglei Yang [3,*] and Jooheon Kim [1,*]

[1] School of Chemical Engineering & Materials Science, Chung-Ang University, Seoul 06974, Korea; mohani@cau.ac.kr
[2] Department of Hydrogen & Renewable Energy, Kyungpook National University, 80 Daehakro, Bukgu, Daegu 41566, Korea
[3] Department of Mechanical and Aerospace Engineering, The Hong Kong University of Science and Technology, Hong Kong SAR, China
* Correspondence: myeongjinkim@knu.ac.kr (M.K.); maeyang@ust.hk (J.Y.); jooheonkim@cau.ac.kr (J.K.); Tel: +82-2-820-5763 (J.K.); Fax: +82-2-812-3495 (J.K.)

Received: 1 May 2020; Accepted: 30 May 2020; Published: date

Supporting Information Contents:

1. Experimental Methods

2. Figures

3. Tables

1. Experimental Methods

1.1. Preparation of SnSe Decorated with Te Nanoneedles

The SnSe crystals used in the present work were supplied in 99.999% purity by Alfa Aesar. The TeO_2 (99%), polyvinylpyrrolidone (PVP, molecular weight ~40,000) and ethylene glycol ($C_2H_6O_2$, 99%) were supplied by Daejung Chemicals & Metals Co., Ltd. The SnSe crystals were ball-milled into small particles using milling machine (HPM-700, Haji Engineering) with zirconia balls in an inert atmosphere (N_2 gas). The rotation speed and milling duration of the planetary mill were set to 150 rpm and 6 h to generate a rolling action of the balls. The resulting SnSe powder (0.282 g) was added to a round-bottomed flask containing 150mL of ethylene glycol and stirred for 10 min. TeO_2 (0.3 g,) and PVP (0.27 g) were then added to the solution, followed by stirring and gradual heating to 433 K. This temperature was then maintained for various reaction times (0, 1, 2, 4, and 6 h) for the optimization of the Te nanoneedle synthesis. The obtained samples are subsequently referred to as Te nanoneedles(x)/SnSe, where '*x*' indicates the reaction time (i.e. x = 0, 1, 2, 4, or 6 h). The resulting product was collected and washed several times with water and ethanol, then dried overnight in a vacuum oven. Finally, the product was pressed under 50 MPa at 823 K for 10 min, yielding pelletized samples.

1.2. Characterization

The crystal structure of the materials was characterized by X-ray diffraction (XRD; New D8-Advance/Bruker-AXS) at 40 mA and 40 kV, with Cu Kα radiation (0.154056 nm) and a scan rate of 1°/s for 2θ ranging from 5° to 70°. The morphologies were observed by field-emission scanning electron microscopy (FE-SEM, SIGMA). The elemental mappings were performed by energy-dispersive X-ray spectroscopy (EDS, NORAN system 7, Thermo Scientific). The binding energy peaks were investigated by X-ray photoelectron spectroscopy (XPS, Thermo U. K. K-alpha) using a monochromatic Al Kα X-ray source (1486.6 eV) and a hemispherical analyzer. The thermal

conductivities were calculated from the formula $\kappa = \alpha \cdot \rho_d \cdot C_P$, where α is the thermal diffusivity, C_P is the specific heat, and ρ_d is the bulk density of the material. The thermal diffusivity was measured using an LFA-457 Nanoflash (NETZSCH) and the specific heat was measured using differential scanning calorimetry (DSC 131 Evo, Setaram Instrumentation). The DSC measurements described the thermal transition behavior of the composites, with the samples heated from −20 to 120 °C at a rate of 10 °C/min in a N_2 atmosphere. A four-point probe method with disk-shaped compressed pellets was used to measure the electrical conductivity. A custom-made device containing a pair of thermocouples and voltmeters was used to measure the Seebeck coefficient, *S*. The measurement was repeated five times for each sample for reproducibility.

2. Figures

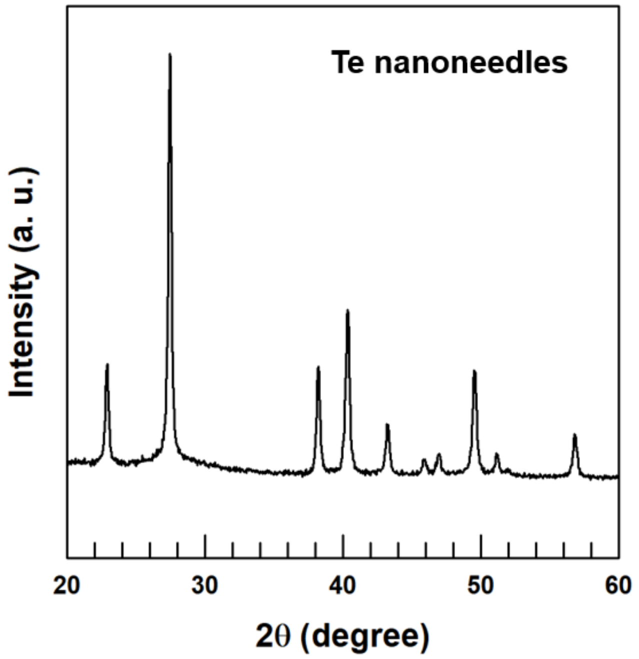

Figure S1. XRD patterns of the Te nanoneedles.

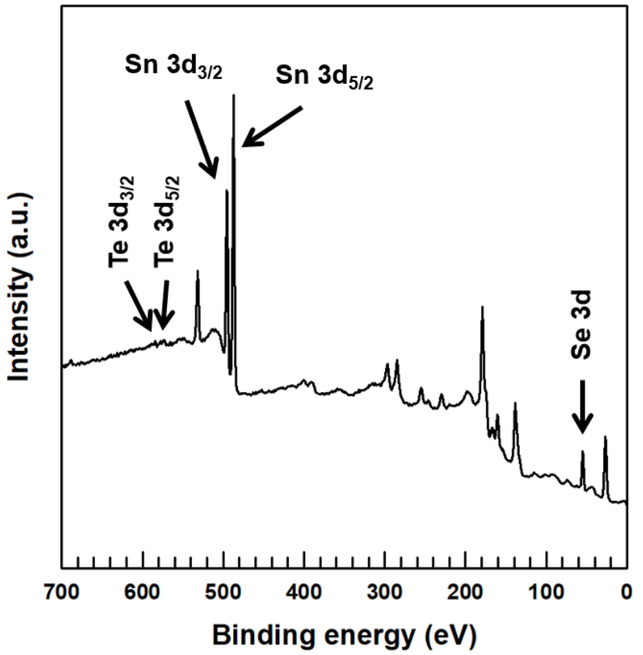

Figure S2. Survey scan XPS spectrum of the Te nanoneedles(4h)/SnSe composite.

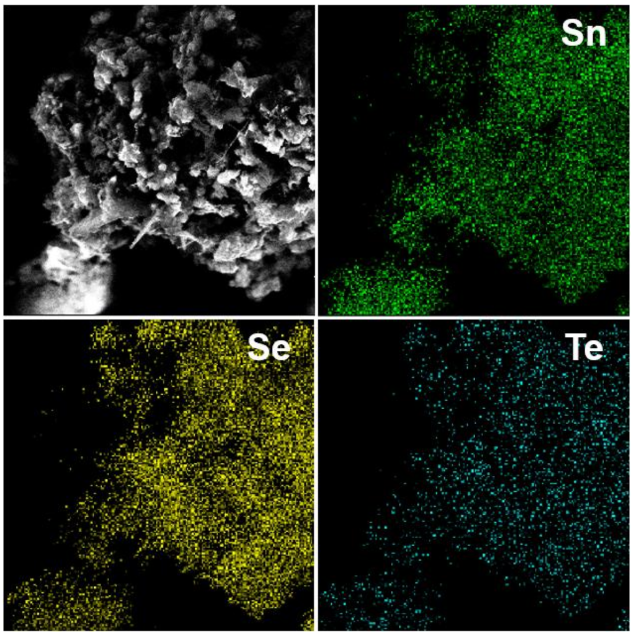

Figure S3. FE-SEM image of the Te nanoneedles(4h)/SnSe composite and the resulting EDS mappings of Sn, Se, and Te atoms.

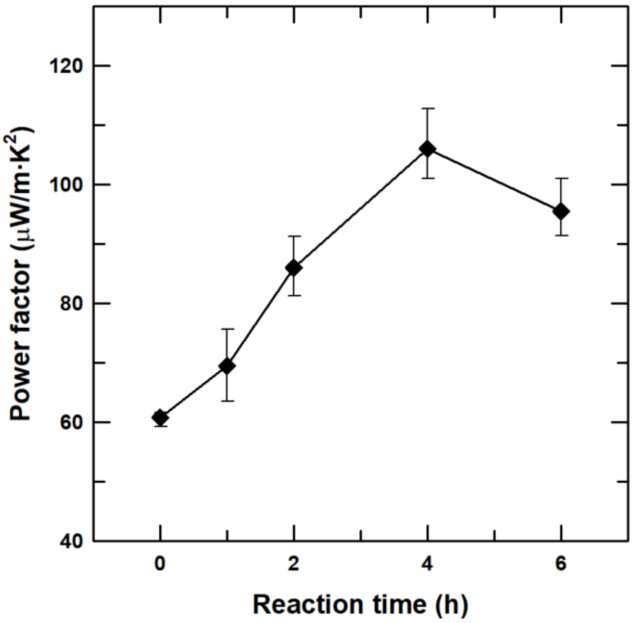

Figure S4. Power factor (σS^2) values of the Te nanoneedles(x)/SnSe composites as a function of reaction time.

© 2020 by the authors. Submitted for possible open access publication under the terms and conditions of the Creative Commons Attribution (CC BY) license (http://creativecommons.org/licenses/by/4.0/).
